# Crystallization of CsPbBr_3_ single crystals in water for X-ray detection

**DOI:** 10.1038/s41467-021-21805-0

**Published:** 2021-03-09

**Authors:** Jiali Peng, Chelsea Q. Xia, Yalun Xu, Ruiming Li, Lihao Cui, Jack K. Clegg, Laura M. Herz, Michael B. Johnston, Qianqian Lin

**Affiliations:** 1 Key Lab of Artificial Micro- and Nano-Structures of Ministry of Education of China, School of Physics and Technology, Wuhan University, Wuhan, P. R. China; 2 Department of Physics, University of Oxford, Clarendon Laboratory, Oxford, UK; 3 School of Chemistry and Molecular Biosciences, The University of Queensland, Brisbane, Australia

**Keywords:** Electronic devices, Electronics, photonics and device physics

## Abstract

Metal halide perovskites have fascinated the research community over the past decade, and demonstrated unprecedented success in optoelectronics. In particular, perovskite single crystals have emerged as promising candidates for ionization radiation detection, due to the excellent opto-electronic properties. However, most of the reported crystals are grown in organic solvents and require high temperature. In this work, we develop a low-temperature crystallization strategy to grow CsPbBr_3_ perovskite single crystals in water. Then, we carefully investigate the structure and optoelectronic properties of the crystals obtained, and compare them with CsPbBr_3_ crystals grown in dimethyl sulfoxide. Interestingly, the water grown crystals exhibit a distinct crystal habit, superior charge transport properties and better stability in air. We also fabricate X-ray detectors based on the CsPbBr_3_ crystals, and systematically characterize their device performance. The crystals grown in water demonstrate great potential for X-ray imaging with enhanced performance metrics.

## Abstract (web summary)

Perovskite single crystals are commonly grown in organic solvents, which require relatively high temperature condition. Here, the authors develop a low-temperature crystallisation strategy to grow CsPbBr_3_ single crystals in water with improved charge transport properties and stability.

## Introduction

Metal halide perovskites (MHPs) have emerged as a set of novel semiconductors for next-generation, solution-processed optoelectronic devices, e.g., photovoltaics^1–3^, light-emitting diodes^4^, lasers^5^, and photodetectors^6,7^. The success and rapid progress of perovskite optoelectronics mainly benefit from their facile fabrication, chemical versatility, and excellent optoelectronic properties, such as direct and tunable bandgaps, high and balanced carrier mobility, long carrier lifetime and diffusion lengths, high absorption coefficients, and low recombination rates^8,9^. Furthermore, MHPs can be easily processed with low-cost and low-energy embedded techniques to form quantum dots (QDs), thin films, and large single crystals (SCs). In particular, perovskite single crystals are free of grain boundaries and possess better ambient stability, fewer defects, and longer diffusion length than polycrystalline thin films. Perovskite SCs can also serve as an ideal platform to investigate their intrinsic properties and have vast potential for fabricating optoelectronic devices with state-of-the-art performance metrics^10^. Hence, the growth of perovskite SCs has attracted considerable attention in the past few years, and various growth techniques have been developed for specific MHPs.

Several solution-based approaches have been reported to grow centimeter-scale perovskite SCs, including solution temperature-lowering (STL) method^11^, inverse-temperature crystallization (ITC)^12–14^, and antisolvent vapor-assisted crystallization (AVC)^15^. In principle, all of these methods grow SCs by slowly reducing the solubility of the target perovskites in a precursor solution. These strategies have successfully prepared both organic-inorganic hybrid perovskite SCs, e.g., CH_3_NH_3_PbX_3_ and H_2_NCHNH_2_PbX_3_ and inorganic CsPbBr_3_ SCs. Rakita et al. proposed two simple solution-based methods to grow CsPbBr_3_ from a precursor solution, i.e., antisolvent-vapor saturation treated with acetonitrile or methanol and concentrating the saturated CsPbBr_3_ solution via heating^16^. Meanwhile, Dirin et al. presented a simple and fast route to grow CsPbBr_3_ SCs using the ITC method under ambient atmosphere^17^. Furthermore, Saidaminov et al. reported a modified ITC method that includes a two-step heating process and successfully obtained relatively large CsPbBr_3_ SCs, reaching 3–5 mm^18^. To further improve the quality of CsPbBr_3_ SCs, Zhang et al. modified the ITC method with additives at a reduced temperature. However, this method reduced the crystal growth rate and required almost 10 days to complete the crystallization procedure^19^. Unfortunately, single crystals growth with the ITC methods suffer from solvent corrosion, deteriorating surface morphology and crystalline quality^20^. Recently, Yao and co-authors reported a new strategy, which introduced silicon oil to extract solvent and assist the low-temperature crystallization (LTC). The obtained perovskite SCs presented remarkably reduced defect density and improved crystal quality^21^. However, the residual silicon oil could potentially contaminate the surface of perovskite SCs, and this technique requires an extremely stable growth environment.

To solve the aforementioned issues, we propose a novel strategy to grow large CsPbBr_3_ SCs in water with the presence of hydrobromic acid (HBr). The water-based solution growth of large two-dimensional (2D) layered (NH_4_)_3_Bi_2_I_9_ single crystals have been achieved recently^22^, and Liu et al. reported a fast growth method of CH_3_NH_3_PbI_3_ crystal wafers on aqueous solution surface^23^. However, these crystals possess relatively lower bandgap. CsPbBr_3_ is an inorganic perovskite and has a wider bandgap, which is less sensitive to visible photons, and in principle should give much lower, thermal-generated dark current and noise. It has also been reported that a large number of micro-crystals precipitated when CsBr and PbBr_2_ were mixed and dissolved in hydrobromic acid solution^24,25^. This suggests that CsPbBr_3_ single crystals could be formed in water, and may offer an alternative approach to grow large CsPbBr_3_ crystals at low temperature, but it may require necessary modification and careful control. Hence, we firstly test the solubility of CsPbBr_3_ in water at various temperatures. Then, we modulate temperature to precisely control crystal growth rate. We also introduce a single-crystal seed during the crystal growth process to inhibit the forming of numerous precipitated micro-crystals. In addition, water as an environmentally sustainable, green-solvent is favorable for fabrication. Furthermore, we systematically characterize the crystal structure and optoelectronic properties, and also compare water-grown SCs with their counterparts grown in dimethyl sulfoxide (DMSO). We also fabricate prototype X-ray detectors based on the CsPbBr_3_ single crystals to further evaluate the impact of single-crystal quality on device performance.

## Results

**Fig. 1:**
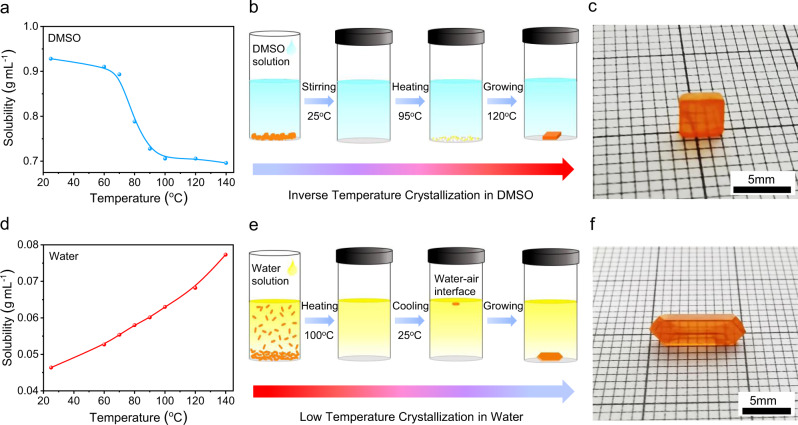
Preparation of CsPbBr_3_ single crystals. **a** Temperature-dependent solubility of CsPbBr_3_ in DMSO. **b** Schematic illustration of the inverse-temperature crystallization in DMSO. **c** Optical photo of the as-prepared CsPbBr_3_ SC. **d** Temperature-dependent solubility of CsPbBr_3_ in water. **e** Schematic illustration of the low-temperature crystallization in water. **f** Optical photo of the as-prepared CsPbBr_3_ SC.

It has been well recognized in the field that the optimal solvents to grow SCs via the ITC method are gamma-butyrolactone (GBL) and dimethylformamide (DMF) for I^−^ and Br^−^ based organic-inorganic hybrid perovskites, respectively^26^. However, the best solvent to grow inorganic CsPbBr_3_ SCs is DMSO, as the solubility of CsPbBr_3_ in DMF has relatively weak temperature dependence. Based on the phase diagram of CsBr:PbBr_2_ compositions^27^, it is challenging to prepare phase-pure CsPbBr_3_ crystals from DMSO, since either the CsBr-rich Cs_4_PbBr_6_ or PbBr_2_-rich CsPb_2_Br_5_ will form if it deviates from a perfect 1:1 CsBr:PbBr_2_ ratio^16–18^. This can be attributed to the huge difference in solubility of CsBr and PbBr_2_ in DMSO, which leads to the precipitation of either CsBr- or PbBr_2_-rich compounds alongside the desired CsPbBr_3_ perovskite^16^. Typically, only upon an increase of the PbBr_2_ fraction to form CsBr:PbBr_2_ (1:2) solution, CsPbBr_3_ crystals can dominate the product^18^. Figure 1a presents the temperature-dependent solubility of CsPbBr_3_ in the optimized CsBr:PbBr_2_ (1:2) DMSO precursor solution^17^. It is clear that the solubility presents a dramatic drop at 70–100 °C from 0.9 g mL^−1^ to 0.7 g mL^−1^. Figure 1b schematically illustrates the ITC growth method in DMSO. It is worth noting that numerous CsPb_2_Br_5_ precipitates formed once the temperature reached 95 °C^18^. Hence, we introduced an extra filtering step and grew pure CsPbBr_3_ SCs at 120 °C with a small CsPbBr_3_ crystal as seed. Figure 1c displays a typical optical photo of the obtained CsPbBr_3_ crystals grown in DMSO, which have a rectangular prism habit with the size of >3 mm. However, it is not trivial to obtain phase pure large CsPbBr_3_ crystals with this method, and it is particularly hard to hinder the formation of additional CsPb_2_Br_5_ crystals on the surface of the obtained CsPbBr_3_ crystals due to the imbalanced stoichiometry.

In contrast, we found the solubility of CsPbBr_3_ in water with the presence of HBr acid decreases with the decrease of temperature gradually, as shown in Fig. 1d. Hence, we suspect CsPbBr_3_ SCs can be grown by cooling down the temperature of water solution. Different from the heating up process in the DMSO method, the crystal structure and composition of the products grown in water can be precisely controlled by adjusting the ratio of CsBr and PbBr_2_ (Supplementary Fig. 1a). Pure CsPbBr_3_ phase can be stabilized when the ratio of CsBr:PbBr_2_ is ≥ 1:1. Otherwise, CsPb_2_Br_5_ can form easily with the addition of excessive PbBr_2_ (Supplementary Fig. 1b, c). We also analyzed the composition of the obtained products with energy dispersive spectroscopy (EDS) (Supplementary Fig. 2), and confirmed the results measured via XRD. Our finding is also consistent with previous reports^28,29^. Hence, we choose CsBr:PbBr_2_ = 1:1 as the optimal precursor composition to grow CsPbBr_3_ SCs in water, and schematically display the LTC method in Fig. 1e. Interestingly, unlike the DMSO technique, the nucleation of CsPbBr_3_ crystal seed appears at the water-air interface (Supplementary Fig. 3). High-quality CsPbBr_3_ SCs can be grown when single CsPbBr_3_ crystal seeds are placed on the bottom of the containers. Figure 1f presents a typical optical photograph of the obtained CsPbBr_3_ SCs from water, which have a smoother surface and different rhombohedral habit, compared with the CsPbBr_3_ SCs from DMSO. The slow cooling rate can provide a quasi-equilibrium between the dissolution and crystallization processes during the growth of high-quality SCs.

Interestingly, the SCs grown in DMSO and in water possess different stability in ambient environment (25 °C, 40% RH). We found that the surface of CsPbBr_3_ SCs obtained from DMSO can form white grains and the surface morphology deteriorated noticeably after exposure in air (20 °C, 40% RH) within 2 min, as shown in Supplementary Fig. 4. After 1 h, the surface of the DMSO-grown crystals was covered with white grains completely. We suspect the fresh prepared DMSO-grown crystals still contain tiny amount of solvent residual, which may have strong interaction with the water molecules in humid air and result poor surface morphology and white grains. It is worth to note that we did not rinse/clean any of the single crystals after growth, and the formation of the surface white grains might be related to this specific growth protocol from DMSO. However, the water-grown crystals do not have any problem during the drying process. To prove this point, we have also prepared and dried DMSO-grown single crystals in glovebox to get rid of the influence of water. We have successfully obtained decent crystals with smooth and reflective surface. Unfortunately, these DMSO-grown crystals can still form white grains on the surface after storage in humid air for one week, as shown in Supplementary Fig. 5. In contrast, the CsPbBr_3_ SCs grown in water can be stored in air for a long time with negligible degradation. The reason is properly due to the strong interaction between lead halides (PbX_2_) and DMSO, which can easily form PbX_2_(DMSO) complex^30^. Furthermore, we characterize the surface morphology with optical microscopy and scanning electron microscopy, and found numerous micro-crystals formed on the surface of the CsPbBr_3_ SCs grown in DMSO (Supplementary Fig. 6). In addition, we also evaluated the elemental composition of these two kinds of SCs with EDS both at the surface and in bulk (Supplementary Fig. 7). The SEM and EDS results suggest that the surface of the CsPbBr_3_ SCs grown in DMSO degraded a lot during their exposure in air. However, the surface of the CsPbBr_3_ SCs grown in water exhibited superior stability to humidity. Furthermore, we polished the single crystals grown in DMSO, compared the optical photos of the as-prepared DMSO-grown, polished DMSO-grown, and as-prepared water-grown single crystals in Supplementary Fig. 8. The polished single crystals exhibited very reflective and smooth surface, and have similar transparency as the water-grown crystals, suggesting the degradation of the DMSO single crystals mainly occurred on the surface. In addition, we also tested and compared their thermal stability via thermal gravimetric analysis (TGA) as shown in Supplementary Fig. 9. All of the CsPbBr_3_ SCs showed relatively high decomposition temperatures of >600 °C, which is in line with the literature reports^18,31^.

**Fig. 2:**
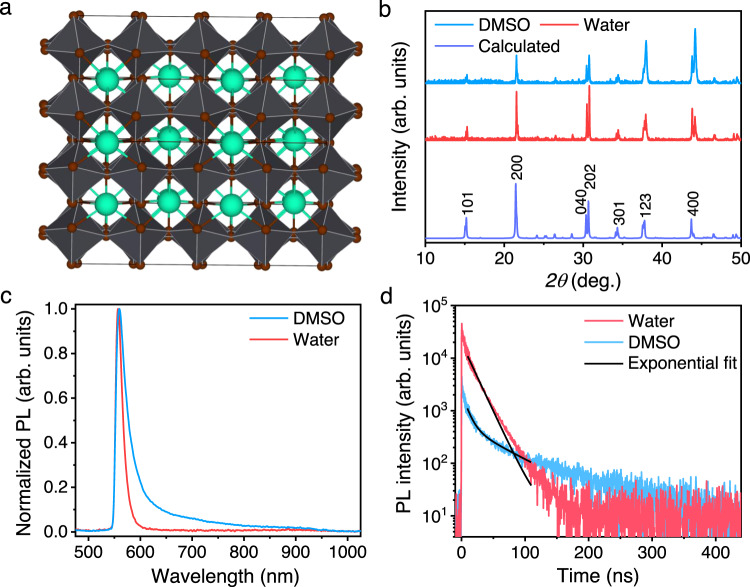
Characterization of CsPbBr_3_ single crystals. **a** Illustration of the crystal structure of CsPbBr_3_, **b** Comparison of the powder X-ray diffraction (XRD) of the DMSO and water grown SCs and the predicted pattern simulated from single-crystal diffraction. **c** Photoluminescence spectra. **d** Time-resolved PL decay of CsPbBr_3_ SCs grown in DMSO and water.

Having obtained decent SCs both from the ITC method in DMSO and the LTC method in water, their structural and optoelectronic properties were systematically characterized and compared. We first determined their powder X-ray diffraction (XRD) patterns. The powder XRD of both is consistent with phase pure materials and the expected orthorhombic (*P*nma) symmetry^32^. Figure 2a depicts the crystal structure, and Fig. 2b compares the powder x-ray diffraction patterns of these two crystals with the simulated pattern. Single-crystal XRD measurements confirmed both crystals were the same phase, but had markedly different habits. Face-indexing shows that the face with the largest surface area in the DMSO-grown crystals is the (100) face while the (001) is the largest face in the water grown crystals, as shown in Supplementary Fig. 10. The water-grown crystals additionally have four (210) faces present which are not present in the DMSO-grown sample; the (100) face is not present in the water-grown crystals.

Figure 2c shows the photoluminescence (PL) spectra of these two types of CsPbBr_3_ SCs. Compared with conventional PL spectra obtained from perovskite thin films, the PL spectra of SCs are distorted, which has been reported previously and attributed to the self-absorption of the short-wavelength photons^33,34^. However, these two kinds of SCs present different full-width-at-half-maximum (FWHM). The narrower PL peak of the SCs grown in water indicates lower energetic disorder and carrier scattering. Moreover, we also determined the charge-carrier lifetimes of these two SCs with time-correlated single photon counting (TCSPC) technique, shown in Fig. 2d. The decay of time-resolved PL is mainly governed by monomolecular charge-carrier recombination with impurities that act as charge traps under the low excitation fluence^35^. Interestingly, the CsPbBr_3_ SCs grown in water present a few times higher TCSPC counts at the beginning of the PL decay, implying much higher PL intensity. We also performed a fluence-dependent PL measurement as shown in Supplementary Fig. 11, which also proved the higher PL intensity from the water-grown crystals. Furthermore, the carrier lifetime can be easily fitted with an exponential decay and resulted a value of ~17 ns. However, the SCs grown with DMSO exhibited two carrier lifetimes (9 ns and 64 ns). The short lifetime in the initial stage of the PL decay was attributed to the monomolecular recombination in the bulk of SCs, and the longer lifetime in the tail may have the contribution from the surface recombination caused by the surface degradation.

We then characterized the relative permittivity of the obtained two types of SCs. The dielectric response was recorded within the frequency range of 20 Hz to 200 kHz, as shown in Supplementary Fig. 12. Both SCs possess almost identical permittivity of ~22 at the high-frequency regime. However, the value increases dramatically with the decrease of frequency, especially for the SCs grown in DMSO. Conventionally, the giant dielectric constants at low frequency are mainly caused by the ionic migration and interface polarization^36^. The ion movement can be slowed down or frozen with the decrease of temperature (Supplementary Fig. 12a, b) to some degree. On the other hand, illumination can generate charge carriers and promote ionic migration^37^. Therefore, the dielectric constants can be enhanced by a few times (Supplementary Fig. 12c, d). However, both the temperature-dependent dielectric response and light-induced permittivity suggest that the SCs grown in water have less ionic feature and therefore could be more stable under electrical poling.

**Fig. 3:**
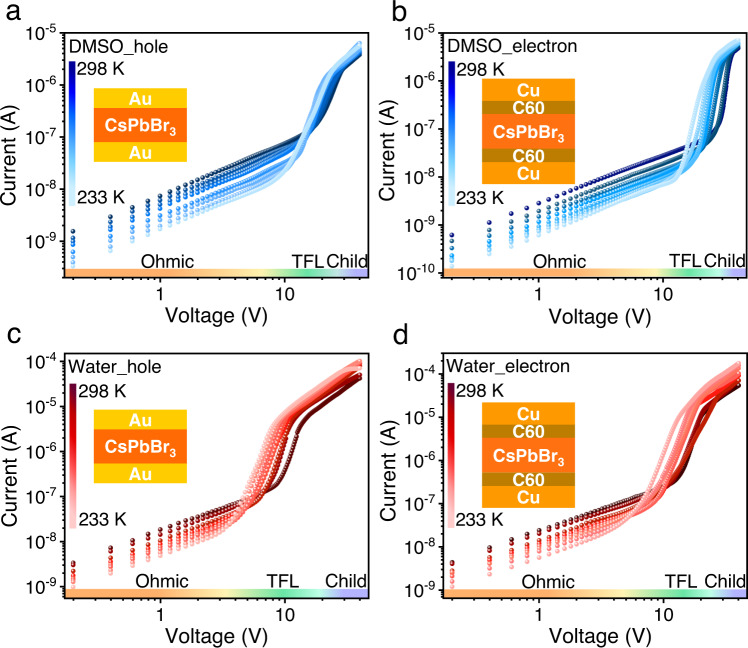
Electrical properties determined by SCLC. Temperature-dependent *J–V* characteristics of **a** hole-only and **b** electron-only devices of CsPbBr_3_ SCs grown in DMSO, and **c** hole-only and **b** electron-only devices of CsPbBr_3_ SCs grown in water. A linear ohmic regime (yellow) is followed by the trap-filled regime, marked by a steep increase in current (green), then followed with Child’s regime (purple). The insets depict the device architectures.

Having the knowledge of dielectric constants, we turn to the determination of electrical properties of these SCs via the space-charge-limited current (SCLC) technique - a commonly used method in the field of semiconductors. Figure 3 presents the temperature-dependent SCLC measurements of the hole-only and electron-only devices based on these two types of SCs. The conductivity, trap density, and charge-carrier mobility can be extracted by fitting the current density–voltage (*J–V*) characteristics. Apparently, the conductivity decreased with the decrease of temperature in the Ohmic regime, and the SCs turned to be more conductive in the Child regime when they are cooled down. Meanwhile, the trap filling regime slightly shifted to low-electric field with decreasing temperature, indicating a decrease of trap density. It is worth noting that the SCLC method can sometimes slightly overestimate the mobility value due to the disturbance of the ion migration at high bias voltage. Therefore, we tested all the SCs many times to minimize the measurement error, and Supplementary Fig. 13 shows the reproducible *J–V* curves obtained from multiple devices with the same device structure. We also tested the SCLC devices with various scan rates and scan polarity as shown in Supplementary Fig. 14. The perovskite-based devices do present scan rate dependence, particularly at high bias voltage. With the increase of scan rates, the current decreased slightly at high voltage. We also tested the device with the same scan rates for several times and found that the current can be stabilized after one or two scans (Supplementary Fig. 14b). The devices did not behave much scan polarity dependence, due to the symmetric device structure, which is consistent with the recent report on perovskite SCLC^38^. Hence, the main error should come from the different scan rates. Supplementary Fig. 15 and Supplementary Table 1 systematically compares the change of these electrical properties versus temperature. Both of two sets of SCs showed similar trends, but the SCs grown in water exhibit higher conductivity and mobility. In particular, the hole and electron carrier mobilities (*μ*_h_ and *μ*_e_) of CsPbBr_3_ SCs grown in water were calculated to be 128 and 160 cm^2^ V^−1^ s^−1^, respectively, which is much higher than those of CsPbBr_3_ SCs grown in DMSO (12.8 and 28.5 cm^2^ V^−1^ s^−1^), Despite the large errors existed at room temperature, the improved charge-carrier mobility based on the LTC method should be valid, considering employing the same technique. In addition, the mobility of the SCs grown in water increased dramatically with cooling, which is consistent with the reported Fröhlich interaction limited mobility at room temperature^39^. Furthermore, we also tested these SCs with optical-pump-THz-probe spectroscopy (OPTPS) with a reflection geometry at room temperature (Supplementary Fig. 16), resulting in charge-carrier sum mobility values of 9.3 cm^2^ V^−1^ s^−1^ and 5.3 cm^2^ V^−1^ s^−1^ for the SCs grown in water and DMSO, respectively. The smaller values obtained from the optical probe are suspected to be affected by the surface degradation, as photogeneration of carrier during the OPTPS measurements mainly occurs near the surface of SCs.

The optoelectronic properties of the obtained CsPbBr_3_ SCs were compared with those for other reported CsPbBr_3_ thin films and CsPbBr_3_ SCs prepared with other methods, as shown in Table 1. It is obvious that the CsPbBr_3_ SCs grown with our LTC method in water possess enhanced charge-carrier mobility and reduced trap density, indicating improved crystal quality of the CsPbBr_3_ SCs.

**Table 1:** Comparison of the optoelectronic properties of CsPbBr_3_ films and SCs prepared with various techniques.

CsPbBr_3_ perovskite	Method	Lifetime (ns)	Trap density (cm^−3^)	Mobility (cm^2^ V^−1^ s^−1^)	Diffusion length (μm)	References
			hole/electron	hole/electron	hole/electron	
Polycrystalline films	Spin-coating	6.9	3.1 × 10^12^	9.27	–	^45^
polycrystalline films	Mist deposition	–	1.3 × 10^12^	13	–	^46^
Monocrystalline film	Hot-pressing	34	1.5 × 10^10^	38	–	^31^
Monocrystalline film	VPE^a^	10.5	1.5 × 10^12^	518	–	^47^
Monocrystalline film	SLAVC^b^	7.6	1.8 × 1 0^10^/1.3 × 10^10^	1770/910	4.2/5.9	^48^
Nanowires	Colloidal	3.55	10^14^–10^15^	–	–	^49^
Single crystal	ITC^c^	233	4.2 × 10^10^/1.1 × 10^10^	11/52	2.5/5.5	^18^
Single crystal	Bridgman	10.5	1.9 × 10^9^	2000/2300	9.5/10.9	^50^
Single crystal	Bridgman	10.9	6.3 × 10^10^	11.6	–	^51^
Single crystal	VSA^d^	30				^16^
Single crystal	Antisolvent vapor	6.8				^52^
Single crystal	Antisolvent vapor	6.8	2.8 × 10^10^	13.6		^53^
Single crystal	AVC^e^			143		^54^
Single crystal	Vapor diffusion	2.3 × 10^3^/7.1 × 10^3^ ^g^		0.05/0.5	1.7/13	^55^
Single crystal	ITC^c^	6.2	7.6 × 10^10^/7.1 × 10^10^	12.8/28.5	0.47/0.68	This work
Single crystal	LTC^f^	16.8	1.7 × 10^10^/2.2 × 10^10^	128/160	2.35/2.63	This work

^a^Vapor-phase epitaxial.

^b^Space-limited antisolvent vapor-assisted crystallization.

^c^Inverse-temperature crystallization.

^d^Slow vapor saturation of an antisolvent.

^e^Antisolvent vapor-assisted crystallization.

^f^Low-temperature crystallization in water.

^g^Hole/electron lifetime measured with time-resolved microwave conductivity.

**Fig. 4:**
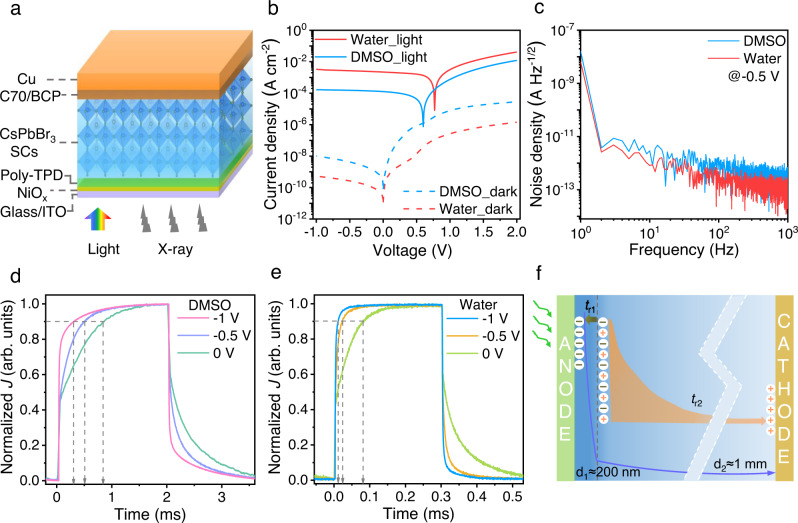
Device performance of CsPbBr_3_ SCs based photodetectors. **a** Schematic illustration of the device structure of CsPbBr_3_ SCs based photodetectors. **b** *J–V* curves measured in the dark and under 100 mW cm^−2^ white light illumination. **c** Noise current spectra. Temporal response of the devices based on SCs grown in **d** DMSO and **e** water. **f** Schematic illustration of the surface generation and imbalanced charge transport of the SCs based devices.

Having a good understanding of the fundamental properties, we now move on to the device consideration. Figure 4a schematically illustrates the device architecture, where SCs are sandwiched between an anode and a cathode, with additional hole and electron transport layers, respectively. The current density–voltage (*J–V*) curves of the CsPbBr_3_ SCs based photodetectors exhibited rectifying characteristics, as shown in Fig. 4b. The photodetectors based on SCs grown in water presented extremely low dark current density of 0.6 nA cm^−2^ @−1 V, which was almost an order of magnitude lower than the devices based on the DMSO method. Moreover, the devices based on the water-grown SCs also showed more than one order of magnitude higher photocurrent than their counterparts under the same irradiation, indicating better charge extraction given larger mobility and similar photogeneration. Figure 4c compares the noise density spectra recorded from these two types of devices in dark. Obviously, the devices based on water grown SCs exhibited less noise, whose value at 1 kHz is around 80 fA Hz^−1/2^. The noise spectra of these two types of devices also tested under 500 Hz modulated weak light illumination with various bias voltages (Supplementary Fig. 17), and showed the same trend. The lower noise density and the higher open-circuit voltage of ~0.77 V (Fig. 4b), further suggest lower recombination rates within the devices based on low-temperature grown SCs. In addition, the light intensity of the noise measurements was attenuated with neutral density filters, down to ~3 nW cm^−2^. We can clearly distinguish the photocurrent response at 500 Hz from the noise floor (Supplementary Fig. 17a), indicating an impressive suitability for weak light detection. Typical temporal responses of the CsPbBr_3_ SCs based detectors were tested with a square-wave modulated 528 nm light-emitting diode (LED) as a light source, as displayed in Fig. 4d, e. Prior to all the transient measurements, we also recorded the resistor-capacitance (RC) time constants of the devices, which could significantly influence the transient curves. Supplementary Fig. 18a demonstrates the RC decay curves of the devices based on DMSO-grown and water-grown crystals, responding to the square-wave modulated bias voltage applied on these single-crystal devices. Both of the devices showed a small RC value of <300 ns with a load resistor (*R*_Load_) of 500 Ω. To further explore the influence of the load resistance, we recorded the RC decay curves as a function of load resistance, and show the results in Supplementary Fig. 18b. RC decay time increased almost linearly with the increase of *R*_Load_, suggesting *R*_Load_ should be small (<500 Ω) to gain a high time resolution. It is clear that all the devices presented field-dependent responses, and the devices based on the LTC showed a faster response, compared with the DMSO-grown SCs. Interestingly, all the SCs based photodetectors present two distinct rise times as shown in Figs. 4d and 4e (the same for the fall times). The photocurrent increased immediately to almost half of the response under illumination, on a time scale termed *t*_r1_. Subsequently, the photocurrent increased slowly to its maximum value, on a time scale termed *t*_r2_, which dominates the response time. Supplementary Fig. 19 displays the typical fast response either under modulated LED or a fast nanosecond laser. Apparently, the faster part (*t*_r1_) of the photocurrent rise dynamic lasts less than 500 ns, which could be also limited by the interfaces and the testing circuit. However, the slower part of the response (*t*_r2_) takes more than 50 μs to complete. This phenomenon can be attributed to imbalanced charge transport, as the visible photons are mainly absorbed near the surface of the SCs and generate electron-holes just near the anode. Hence, it only takes a few hundreds of nanometers for holes to be extracted by the anodes, but electrons need to travel the whole depths of the SCs, which have a thickness of ~1 mm (as illustrated in Fig. 4f). In contrast, we can also test the devices with a semi-transparent copper cathode and evaluate the hole transport. Hence, we inferred the transit time from the −3 dB frequency (*f*_−3 dB_) with illumination on the cathode, which can be used to determine the hole mobility. Supplementary Fig. 20a, b present the temperature-dependent frequency response of these two types of photodetectors, and an increased *f*_−3 dB_ has been observed for both devices at low temperature. The calculated temperature-dependent hole mobilities for DMSO and water grown SCs are shown in Supplementary Fig. 20c, d, which is consistent with the SCLC results.

**Fig. 5:**
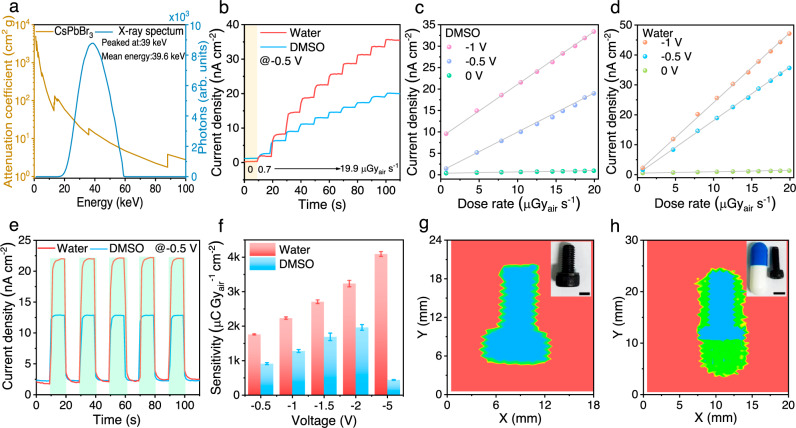
Device performance of CsPbBr_3_ SCs based detectors under X-ray irradiation. **a** Calculated attenuation coefficient of CsPbBr_3_, and the spectra of X-ray source and after attenuation with 1 mm thick SCs. **b**–**d** Comparison of the X-ray generated photocurrent versus various dose rates of the devices based on SCs grown in DMSO and water. **e** Temporal response of these two types of devices to X-ray source switched between 0.7 and 12.5 μGy_air_ s^−1^. **f** X-ray sensitivity of the devices based on SCs grown in DMSO and water, optical photos and corresponding X-ray images of **g** the metal screw and **h** the screw within a capsule (the scale bar is 5 mm).

Finally, we evaluated the device performance under X-ray radiation. Figure 5a displays the attenuation coefficient of CsPbBr_3_ in the energy range of 0–100 keV, and the spectrum of the X-ray source with a tungsten anode operated at 60 kVp (1% ripple) with a set of beam filters (1.4 mm glass, 4.4 mm oil, 1.5 mm Ultem and 0.1 mm Cu). The relative thick (~1 mm) CsPbBr_3_ SCs can effectively absorb X-ray photons. The X-ray dose rates can be easily modulated by tuning the applied current of the X-ray tube, and we recorded the X-ray response of these two types of devices to various dose rates as shown in Fig. 5b. Figure 5c, d presents the X-ray generated current density versus X-ray dose rates with various bias voltages. X-ray sensitivities can be calculated based on the linear fitting of these curves, showing that the sensitivity increased with the increase of bias voltage. Again, the devices based on low-temperature grown SCs exhibited superior sensitivity, which reached 4086 μC Gy_air_^−1^ cm^−2^ at −5 V. This value indicates an impressive sensitivity level, given that it is more than 200 times that for the state-of-the-art commercial α-Se X-ray detectors used for X-ray imaging^40^. The temporal responses to X-rays were recorded by turning the incident X-ray tube current, as shown in Fig. 5e, which revealed decent repeatability for both SCs. Further comparison of the sensitivity of devices based on the optimized CsPbBr_3_ SC grown in water at various bias voltages was shown in Fig. 5f and Supplementary Fig. 21. The high and field-dependent X-ray sensitivity can be attributed to the excellent charge transport of the low-temperature grown SCs and field-dependent charge extraction. We have also observed that these X-ray detectors can work nicely within 10 V bias voltage. With the increase of bias, the X-ray generated current increased significantly. However, the dark current also increased slightly. It is worth to note that our devices are based on a diode structure. The charge transport layers are relatively thin (<100 nm) and could be damaged at high bias voltage. The ionic migration of MHPs can also be accelerated, and causes ion accumulation at the interfaces, which can also deteriorate the high voltage stability. We have also compared the voltage stability of both CsPbBr_3_ single crystals as shown in Supplementary Fig. 22. The dark current of the devices based on DMSO-grown crystals showed a dramatic increase of dark current when the bias voltage reached −5 V. However, the water-grown crystals can survive at > −12 V, and presented improved voltage stability at −10 V both in dark and under X-ray illumination. Compared with the state-of-the-art photoconductors based on (NH_4_)_3_Bi_2_I_9_ single crystals^22^, our X-ray detectors based on the diode architecture exhibited slightly lower sensitivity due to the absence of a gain effect. However, our photodiodes exhibited faster response, reduced dark current (at reverse bias) and less field-dependent sensitivity, suggesting that these devices with diode structure can be driven properly with a reduced bias voltage, and present relatively high sensitivity. To further evaluate the potential for X-ray imaging, the detectors based on water-grown SCs were used, and a screw was *x*, *y*-scanned with the X-ray beam. Figure 5g, h displays the obtained X-ray images of the screw and the screw within a medical capsule shell, respectively. These well-resolved images are the direct results of the high sensitivity, low dark current, and noise of the SCs based devices, and imply great potential of the low-temperature grown SCs for X-ray detection and imaging.

In summary, we developed a LTC method to grow large CsPbBr_3_ SCs in water. Their materials and optoelectronic properties were systematically characterized, and also compared with the CsPbBr_3_ SCs grown with the inverse-temperature method at relatively high temperature in DMSO. We find that the low-temperature grown CsPbBr_3_ SCs exhibit better stability in air, inhibited ion migration, lower defect density, higher charge conductivity, and mobility. The hole and electron mobilities of the water grown CsPbBr_3_ SCs reached 128 and 160 cm^2^ V^−1^ s^−1^, respectively. We also introduced these SCs for photodetection, and the performance of the devices based on the SCs grown in DMSO and water was carefully evaluated and compared. Interestingly, the devices based on the low temperature grown SCs exhibited superior device performance, in terms of sensitivity, response time, dark current, and noise, which was attributed to the much-improved optoelectronic properties of these crystals. More importantly, the device based on water-grown CsPbBr_3_ SCs showed a high X-ray sensitivity of 4086 μC Gy_air_^−1^ cm^−2^, which is 200 times higher than the value of commercial α-Se detectors (20 μC Gy_air_^−1^ cm^−2^). The optimized devices also showed excellent applicability for X-ray imaging, which is promising for their use in diagnostics.

## Methods

### Materials

Lead bromide (PbBr_2_, 99% trace metals basis) and cesium bromide (CsBr, 99%) were purchased from Aladdin. Nickel (II) acetate tetrahydrate (NiC_4_H_6_O_4_·4H_2_O, AR, 99%) was purchased from Aladdin. Poly[bis(4-phenyl)(4-butylphenyl)amine] (Poly-TPD) and fullerene-C70 (C70, >99%) were purchased from Xi’an Polymer Light Technology Corp. Dimethyl sulfoxide (DMSO) was purchased from Sigma-Aldrich. Hydrobromic acid (HBr, 48 wt% water) was purchased from Macklin. Chlorobenzene (C_6_H_5_Cl, AR, 99%) was purchased from Aladdin. All commercial products were used as received.

Growth of the CsPbBr_3_ perovskite SCs in DMSO: The CsPbBr_3_ perovskite SCs were prepared via a modified inverse-temperature crystallization^18^. Typically, CsBr and PbBr_2_ (molar ratio of 1:2) were dissolved in DMSO solvent to form saturated perovskite precursor solution. To completely dissolve CsBr and PbBr_2_, the solution was stirred at room temperature for 2 h, followed by gradually heating to 95 °C. Then, the solution was filtered at 95 °C and a small CsPbBr_3_ crystal was placed at the bottom of the container as seed, and kept the solution in a quiet environment at 95 °C for 3 h. To make the crystals larger, the solution was further heated to 120 °C with a rate of 5 °C per 30 min.

Growth of the CsPbBr_3_ perovskite SCs in water: The CsPbBr_3_ perovskite SCs were grown via gradually decreasing the temperature of the water solution. CsBr and PbBr_2_ (molar ratio of 1:1) were dissolved in HBr solution (48 wt% H_2_O) to form saturated perovskite precursor solution. To guarantee fully dissolution, the solution was stirred at 100 °C for 1 h. Then, the solution was filtrated and transferred to a clean container with a seed crystal placed on the bottom, which was placed on a stable hot plate and gradually cooled with a rate of 2 °C h^−1^ to room temperature. The crystal growth process took ~36 h until large single crystals formed.

Materials characterization: the crystal structure of the perovskite SCs and powders were tested with X-ray diffraction (XRD). Powder XRD patterns were recorded using a Bruker Advance D8 X-ray diffractometer with Cu Kα radiation at 1.5418 Å, scanned from 10° to 50°. Single Crystal X-ray Diffraction data were collected using a Rigaku XtaLAB Synergy-S diffractometer equipped with a Pilatus CdTe 300 K detector and employing monochromated Mo-Kα radiation from a sealed X-ray tube Data was collected at 294 (2) K and solved using SHELXT^41^ with further structural refinements carried out using SHELXL-2018/3^42^ within the Olex2 graphical user interface. Atoms were refined anisotropically. The structure proved to be twinned by a two-fold rotation around the *b-*axis wand the BASF parameter refined to 0.22. Surface morphology and the composition of the perovskite SCs were characterized with Scanning electron microscopy (SEM) images and EDS, using a Zeiss SIGMA field emission scanning electron microscope operated at 5 kV. Thermogravimetry was carried out under a constant flow of nitrogen at 40 mL min^−1^ using a Netzsch STA 449 C thermal analyzer. Each sample was heated from room temperature to 1000 °C under nitrogen with a scan rate of 5 °C min^−1^. Steady-state PL spectra were recorded with a fiber optic spectrometer (Ideaoptics, NOVA-EX) excited with a 445 nm CW laser. The time-resolved photoluminescence (TR-PL) was recorded with a time-correlated single-photon counter (PicoQuant GmbH, TimeHarp 260), excited with a 405 nm picosecond laser (Advanced Laser Diode Systems A.L.S. GmbH, Pilas).

### Mobility measurements

The dielectric constants were calculated based on the capacitance values measured with LCR meter (TH2832, Tonghui). The device under test (DUT) is directly connected with the LCR meter. We chose the *C*_p_-*R*_p_ mode, as the thick single crystal-based devices do present high impedance and relatively small capacitance. Hence, *C*_p_ and *R*_p_ versus frequency can be directly recorded with the LCR meter. The real (*ε*_*r*_) and imaginary (*ε*_*im*_) parts of the dielectric constant can be calculated by the following equations, respectively:

1 $$\varepsilon _{{\rm{im}}} = \frac{d}{{2\pi fAR_{\rm{p}}\varepsilon _0}}$$

2 $$\varepsilon _{\rm{r}} = \frac{{dC_{\rm{p}}}}{{A\varepsilon _0}}$$

where *d* is the thickness of the crystal, *f* is the frequency, *A* is the area, *R*_p_ is the parallel resistance, *C*_p_ is the parallel capacitance, and *ε*_0_ is the vacuum permittivity. The charge-carrier mobilities of the obtained SCs were mainly evaluated with the Space-charge-limited current (SCLC) method. Current as a function of the applied voltage was recorded at room temperature or cooled with liquid nitrogen within a cryostat (HCS621G, Instec), using a B1500A semiconductor analyzer (Keysight). Electrodes were deposited on opposite sides of the sample by consecutive thermal evaporation, with a structure of Cu (100 nm)/C60 (20 nm)/CsPbBr_3_ SCs/C60 (20 nm)/Cu (30 nm) for electron-only devices and Au (60 nm)/CsPbBr_3_ SCs/Au (30 nm) for hole-only devices, respectively. Temperature-dependent conductivity and mobility were calculated based on the fitting of the obtained SCLC curves. Meanwhile, the room temperature mobility of the SCs was also measured via optical-pump-terahertz-probe spectroscopy (OPTPS). The terahertz (THz) pulse was generated by a THz spintronic emitter via spin Hall effect^43^ and the reflected THz pulse from the sample surface was detected by a (110) ZnTe crystal via electro-optic sampling. The SCs were photoexcited by ultrafast (35 fs) laser pulses (pump) at 3.1 eV photon energy (wavelength 400 nm), which generated free charge carriers in the SCs and reduced the reflectivity of the THz signal (probe). The change of the THz reflectivity, i.e., photoconductivity, was recorded as a function of time after photoexcitation at fluence 37 *μ*J cm^−2^. The charge-carrier sum mobility *μ* was extracted from the peak of the photoconductivity decay curve according to the following equation^44^:

3 $$\mu = - \frac{{\varepsilon _0c(n^2 - 1)}}{2}\frac{{A_{{\mathrm{eff}}}hc}}{{Ee\lambda (1 - R_{{\mathrm{pump}}})(1 - T_{{\mathrm{pump}}})}}\left( {\frac{{\Delta R}}{R}} \right)$$

where *ɛ*_0_ is the permittivity of vacuum, *c* is the speed of light, *n* is the refractive index of the SCs, *A*_eff_ is the effective overlapping area between the pump pulse and the THz pulse, *h* is the Planck constant, *E* is the photon energy of the pump pulse with *λ* = 400 nm, *e* is the elementary charge, *R*_pump_ and *T*_pump_ are the reflection and transmission coefficients of the SCs at 400 nm, respectively.

Device fabrication and testing: All the photodetector devices were fabricated based on commercial patterned Indium Tin Oxide (ITO) substrates (Meijingyuan Glass). The fabricated devices have a structure of ITO/NiO_x_/Poly-TPD/CsPbBr_3_ SCs/C70/BCP/Cu. The NiO_x_ precursor solution was prepared by adding 49.75 mg nickel (II) acetate tetrahydrate and 12 μL 2-aminoethanol in 1 mL ethylene glycol monomethyl ether and stirred for 2 h under 70 °C. The obtained NiO_*x*_ precursor was spin-coated on the substrates at 3000 rpm for 30 s in air to form hole transport layer (HTL). The NiO_*x*_-coated substrates were pre-annealed at 150 °C for 5 min on a hot plate, followed with another annealing treatment in a muffle furnace at 300 °C for 60 min. After cooling, the substrates were transferred to a nitrogen-filled glovebox for device fabrication. 20 μL, 15 mg mL^−1^ Poly-TPD chlorobenzene solution were dropped on the NiO_x_ coated ITO substrates, and the CsPbBr_3_ single crystals were immediately placed on Poly-TPD solution coated wet substrates. After heating the devices at 100 °C for 30 min to completely remove the solvent, the adhesive Poly-TPD layer (~80 nm) can effectively glue the single crystals on the ITO electrodes. Then, we evaporated 70 nm C70 and 3 nm BCP under vacuum (~ 4 × 10^−4^ Pa, at a rate of ~0.1 Å s^−1^) as electron transport layers. A 60 nm Cu layer was evaporated with a rate of ~0.5 Å s^−1^ to complete the device fabrication. All devices were stored and measured in air at ~25 °C with a humidity level of ~40% RH. *J–V* characteristics (both in dark and under illumination) of the devices were tested with a Keithley 2400 source meter. Frequency response were tested using 528 nm LEDs (Thorlabs) modulated with an arbitrary wave function generator (Agilent, 33612 A) or a nanosecond OPO laser (LS-2145-OPO-PC, Lotis-tii), and the photocurrent responses of these devices were recorded with a digital storage oscilloscope (LeCroy Waverunner 8254). Noise current was measured with a spectrum analyzer (Keysight N9915A FieldFox) with a current pre-amplifier (Stanford Research System, SR570). The device performance under X-ray irradiation was carried out with a commercially available SPELLMAN XRB011 tube (tungsten anode, 20 W maximum power output) as X-ray source. Photodetectors were placed in a dark lead shielded box to prevent ionizing radiation leakage and block the external visible lights. Semiconductor Analyzer B1500A was used to record the photocurrent of photodetectors under various X-ray doses. The radiation dose rate was carefully calibrated with a standard dosimeter (Suhe Instrument Technology Co. LTD, XH-3525).

## Supplementary information

Supplementary Information

## Data Availability

All relevant data are available from the authors. The X-ray crystallographic coordinates for structures reported in this study have been deposited at the Cambridge Crystallographic Data Centre (CCDC), under deposition numbers 2036635. These data can be obtained free of charge from The Cambridge Crystallographic Data Centre via www.ccdc.cam.ac.uk/data_request/cif.
