# Supplementary material for: Crystallization of CsPbBr3 single crystals in water for X-ray detection
Source: Nat Commun. 2021 Mar 9;12:1531. doi: 10.1038/s41467-021-21805-0 (PMC7943776; doi:10.1038/s41467-021-21805-0)
Supplement: Supplementary file 1 — Supplementary Information [file 41467_2021_21805_MOESM1_ESM.pdf]

Supplementary Information

Crystallization of $CsPbBr_3$ single crystals in water for X-ray detection

Jiali Peng,[1] Chelsea Q. Xia,[2] Yalun Xu,[1] Ruiming Li,[1] Lihao Cui,[1] Jack K. Clegg,[3] Laura M. Herz,[2] Michael B. Johnston,[2] and Qianqian Lin[*,1]

[1] *Key Lab of Artificial Micro- and Nano-Structures of Ministry of Education of China, School of Physics and Technology, Wuhan University, Wuhan 430072, P. R. China*

[2] *Department of Physics, University of Oxford, Clarendon Laboratory, Parks Road, Oxford, OX1 3PU, U.K.*

[3] *School of Chemistry and Molecular Biosciences, The University of Queensland, Brisbane, QLD4072, Australia*

Corresponding Author：

*Email: q.lin@whu.edu.cn

Supplementary Figures

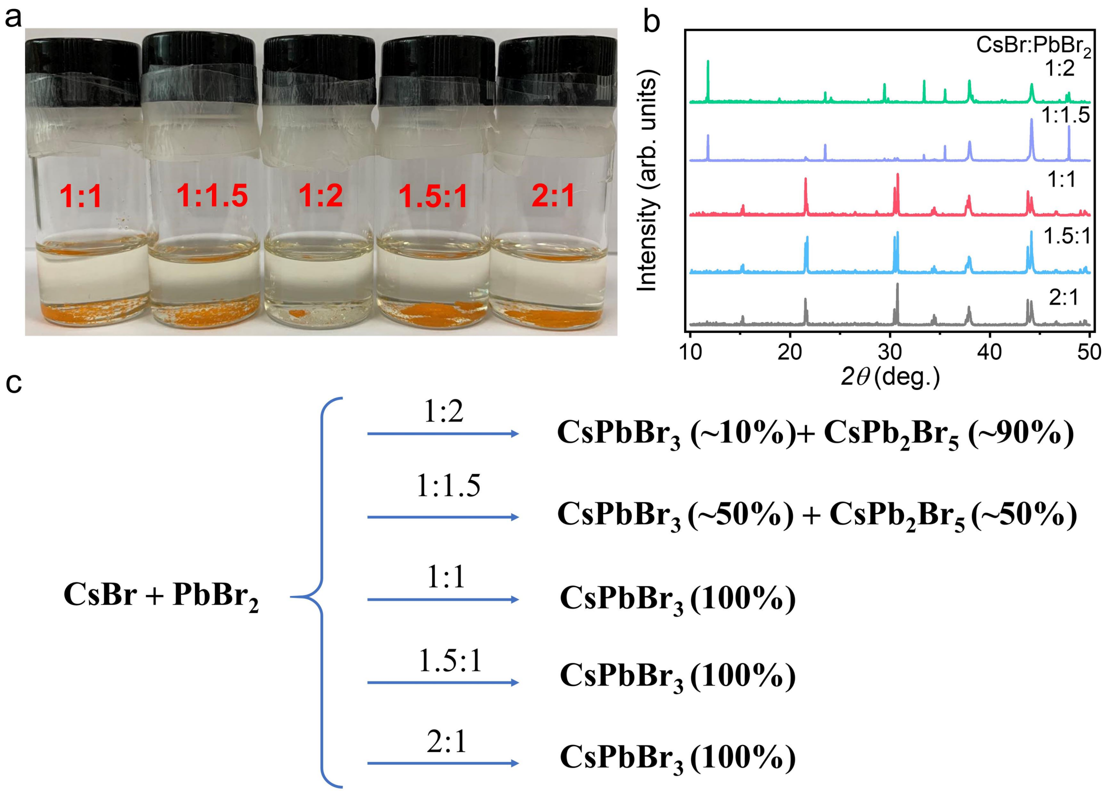

Supplementary Figure 1. (a) Optical photos and (b) powder X-ray diffraction patterns of $CsPbBr_3$ SCs grown in water with various CsBr:$PbBr_2$ ratios. (c) Schematic illustration of the crystallization routes with different precursor composition.

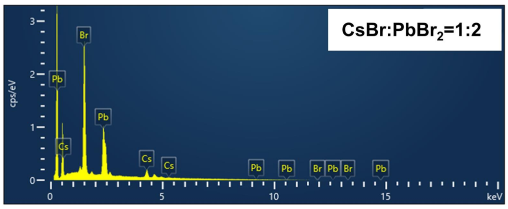

Element	Line style	k ratio	Wt%	Wt% Sigma	Atomic percent
Br	L	0.28092	43.85	0.43	64.48
Cs	L	0.07921	11.6	0.36	10.26
Pb	M	0.26711	44.55	0.48	25.27
Total quantity			100.00		100.00

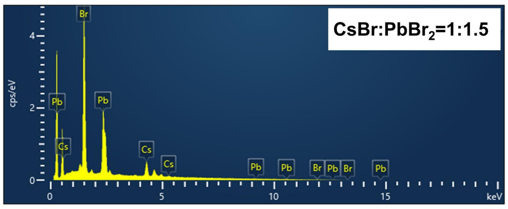

Element	Line style	k ratio	Wt%	Wt% Sigma	Atomic percent
Br	L	0.32576	41.89	0.39	61.75
Cs	L	0.14169	16.43	0.35	14.56
Pb	M	0.31290	41.67	0.45	23.69
Total quantity			100.00		100.00

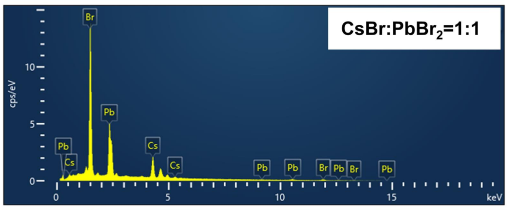

Element	Line style	k ratio	Wt%	Wt% Sigma	Atomic percent
Br	L	0.41983	40.71	0.34	59.27
Cs	L	0.28625	23.69	0.32	20.74
Pb	M	0.36643	25.60	0.40	19.99
Total quantity			100.00		100.00

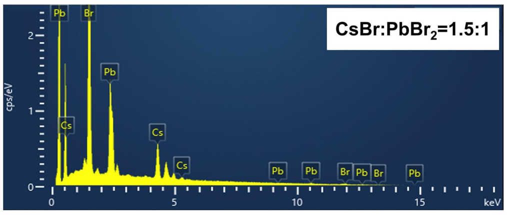

Element	Line style	k ratio	Wt%	Wt% Sigma	Atomic percent
Br	L	0.28184	40.89	0.40	59.52
Cs	L	0.18361	22.83	0.38	20.02
Pb	M	0.24986	36.37	0.48	20.46
Total quantity			100.00		100.00

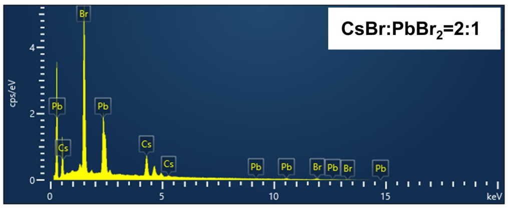

Element	Line style	k ratio	Wt%	Wt% Sigma	Atomic percent
Br	L	0.31265	40.71	0.37	59.49
Cs	L	0.20115	22.54	0.36	19.80
Pb	M	0.28057	36.75	0.44	20.71
Total quantity			100.00		100.00

Supplementary Figure 2. Energy dispersive spectroscopy (EDS) analysis of $CsPbBr_3$ SCs grown with different CsBr:$PbBr_2$ ratios in precursor solution.

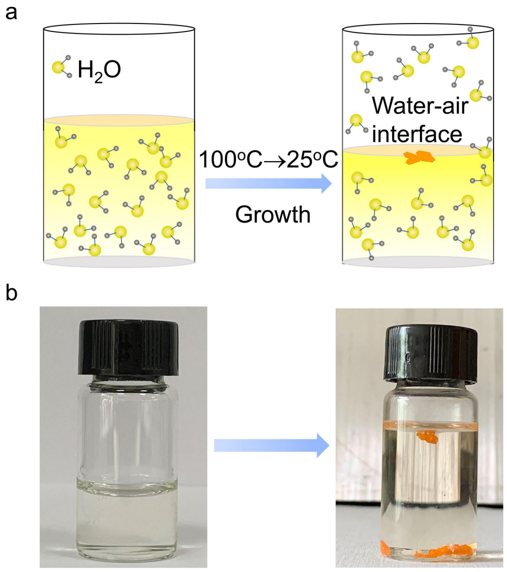

Supplementary Figure 3. (a) Schematic illustration and (b) optical photos of the formation of $CsPbBr_3$ SCs at the water/air interface.

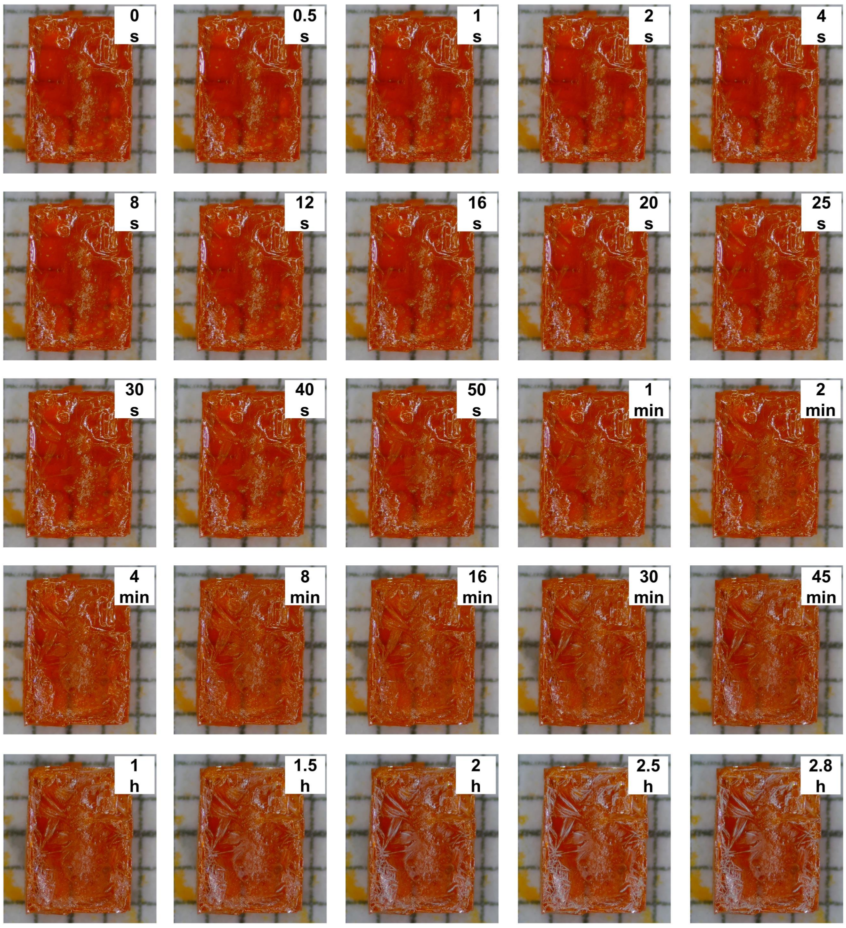

Supplementary Figure 4. Surface morphology evolution of the $CsPbBr_3$ SCs grown in DMSO, recorded with optical microscope.

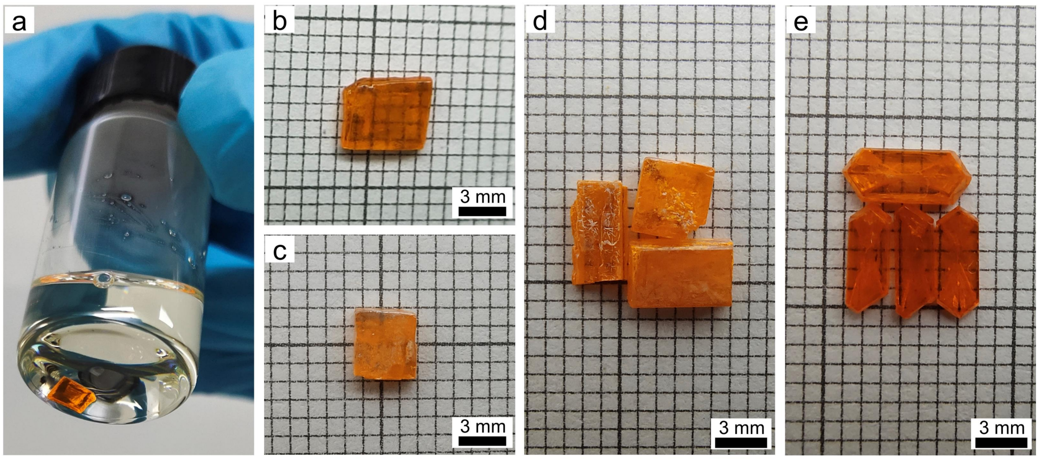

Supplementary Figure 5. Optical photo of (a) DMSO-grown $CsPbBr_3$ SCs in DMSO, comparison of DMSO-grown $CsPbBr_3$ SCs prepared and dried (b) in glovebox and (c) in air, comparison of the long-term air stability of the $CsPbBr_3$ SCs grown (d) in DMSO and (e) in water after exposed in air for one week.

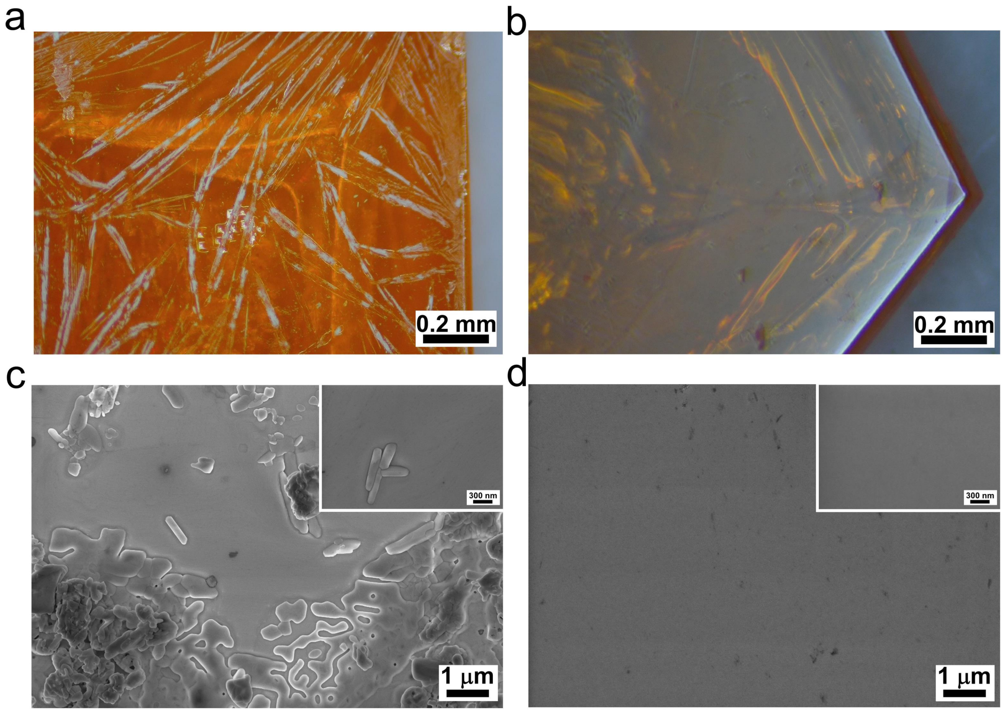

Supplementary Figure 6. Typical optical photos of $CsPbBr_3$ SCs grown in (a) DMSO and (b) water after exposure in air (40% RH) for 3 hours, SEM images the surface morphology of $CsPbBr_3$ SCs grown in of (c) DMSO and (b) water after exposure in air (40% RH) for 3 hours.

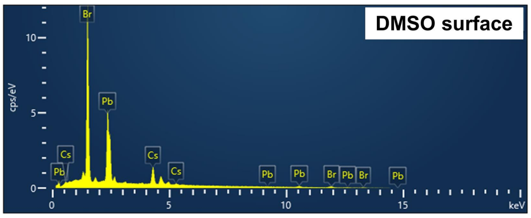

Element	Line style	k ratio	Wt%	Wt% Sigma	Atomic percent
Br	L	0.41314	40.21	0.34	59.97
Cs	L	0.20201	17.53	0.32	15.72
Pb	M	0.42600	42.26	0.40	24.31
Total quantity			100.00		100.00

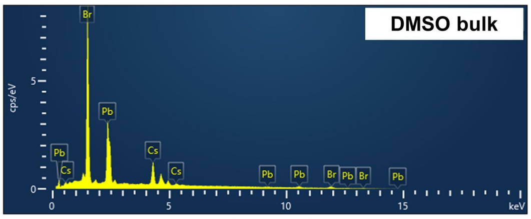

Element	Line style	k ratio	Wt%	Wt% Sigma	Atomic percent
Br	L	0.38699	41.13	0.35	59.91
Cs	L	0.24407	22.37	0.34	19.59
Pb	M	0.34012	36.49	0.42	20.50
Total quantity			100.00		100.00

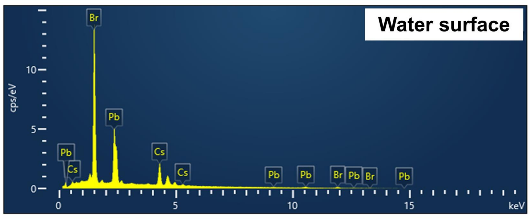

Element	Line style	k ratio	Wt%	Wt% Sigma	Atomic percent
Br	L	0.41983	40.71	0.34	59.27
Cs	L	0.28625	23.69	0.32	20.74
Pb	M	0.36643	35.60	0.40	19.99
Total quantity			100.00		100.00

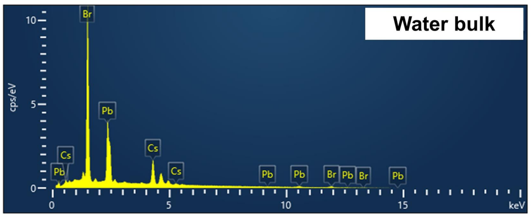

Element	Line style	k ratio	Wt%	Wt% Sigma	Atomic percent
Br	L	0.40892	40.55	0.34	59.11
Cs	L	0.28073	23.74	0.33	20.81
Pb	M	0.35996	35.71	0.40	20.08
Total quantity			100.00		100.00

Supplementary Figure 7. Energy dispersive spectroscopy (EDS) analysis of the surface and bulk composition of $CsPbBr_3$ SCs grown in DMSO and water.

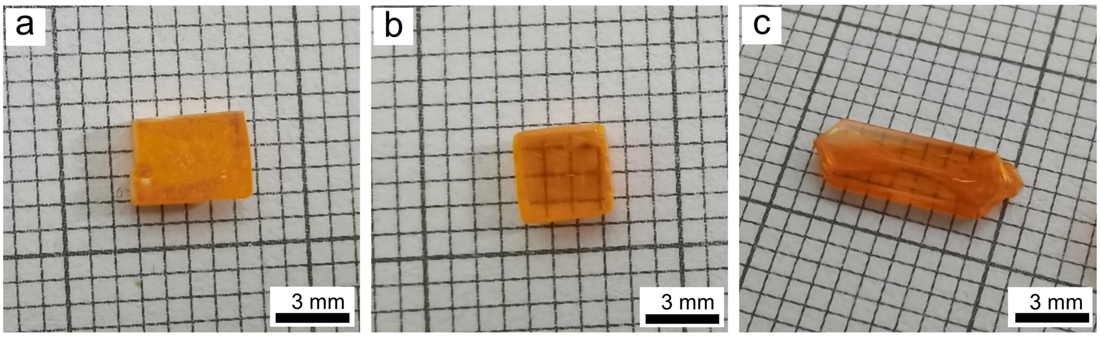

Supplementary Figure 8. Comparison of the optical photos of (a) as-prepared DMSO-grown, (b) polished DMSO-grown and (c) as-prepared water-grown single crystals. The polished DMSO-grown single crystals exhibited very reflective and smooth surface, and have similar transparency as the water-grown crystals.

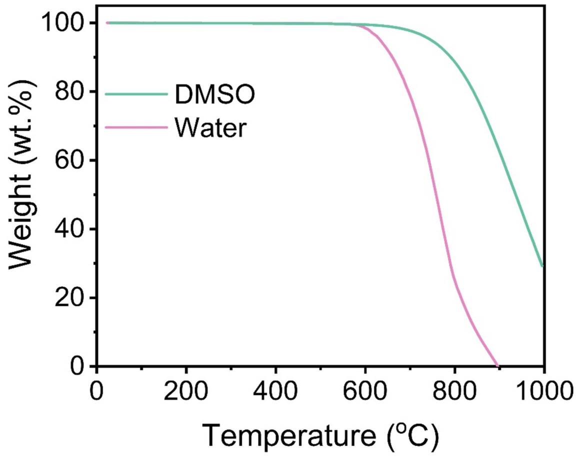

Supplementary Figure 9. Comparison of the thermal gravimetric analysis (TGA) of $CsPbBr_3$ SCs grown in DMSO and water.

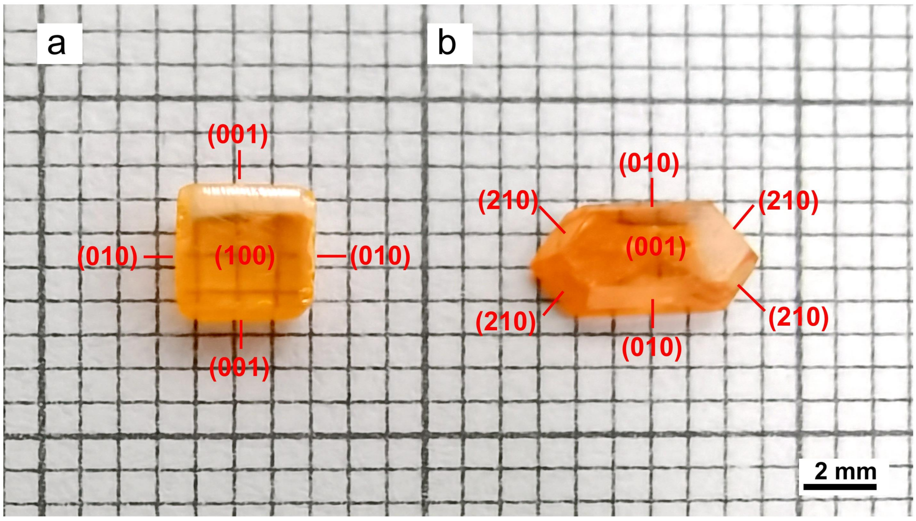

Supplementary Figure 10. Typical optical photos with face-indexing of $CsPbBr_3$ SCs grown in (a) DMSO and (b) water.

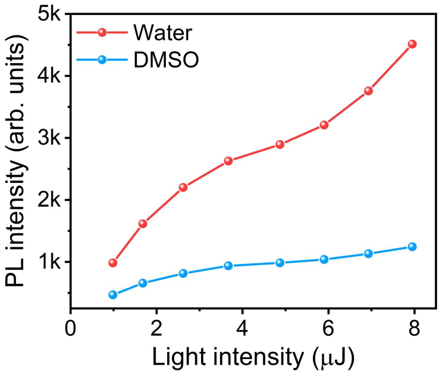

Supplementary Figure 11. Fluence-dependent PL intensity of the DMSO and water-grown $CsPbBr_3$ SCs.

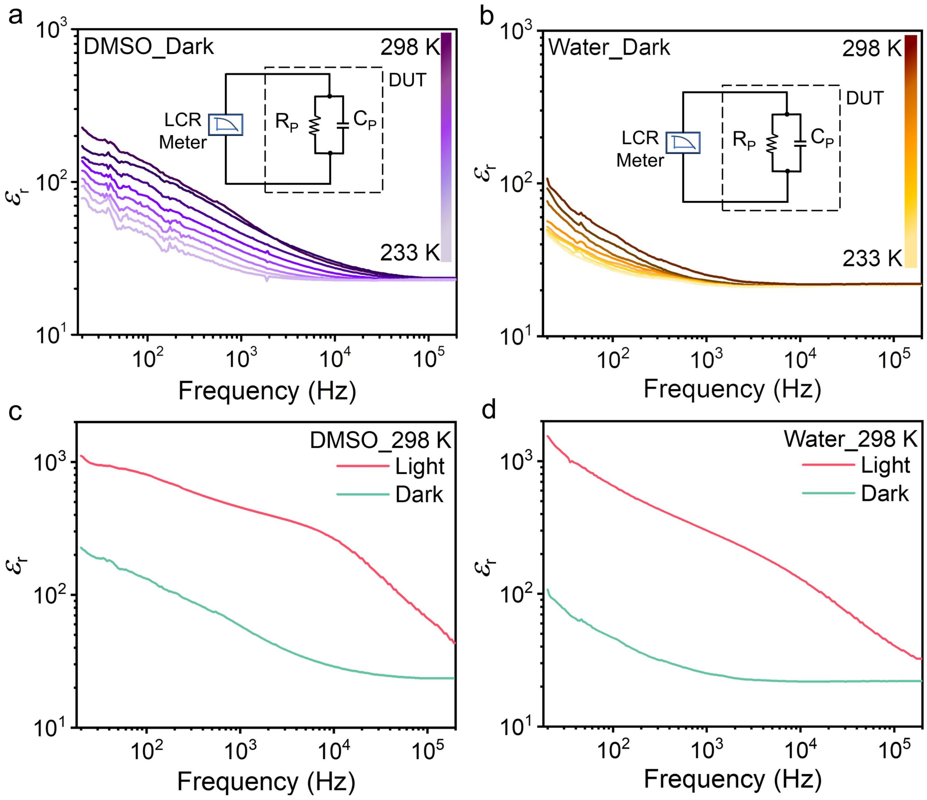

Supplementary Figure 12. Temperature-dependent relative permittivity of the $CsPbBr_3$ SCs grown in (a) DMSO and (b) water, comparison of the permittivity in dark and under 100 mW cm^{-2} white light illumination of the $CsPbBr_3$ SCs grown in (c) DMSO and (d) water. The insets are the equivalent circuits for extracting of the capacitance values, where C_p is the parallel capacitance, R_p is the parallel resistance and DUT is the device under test.

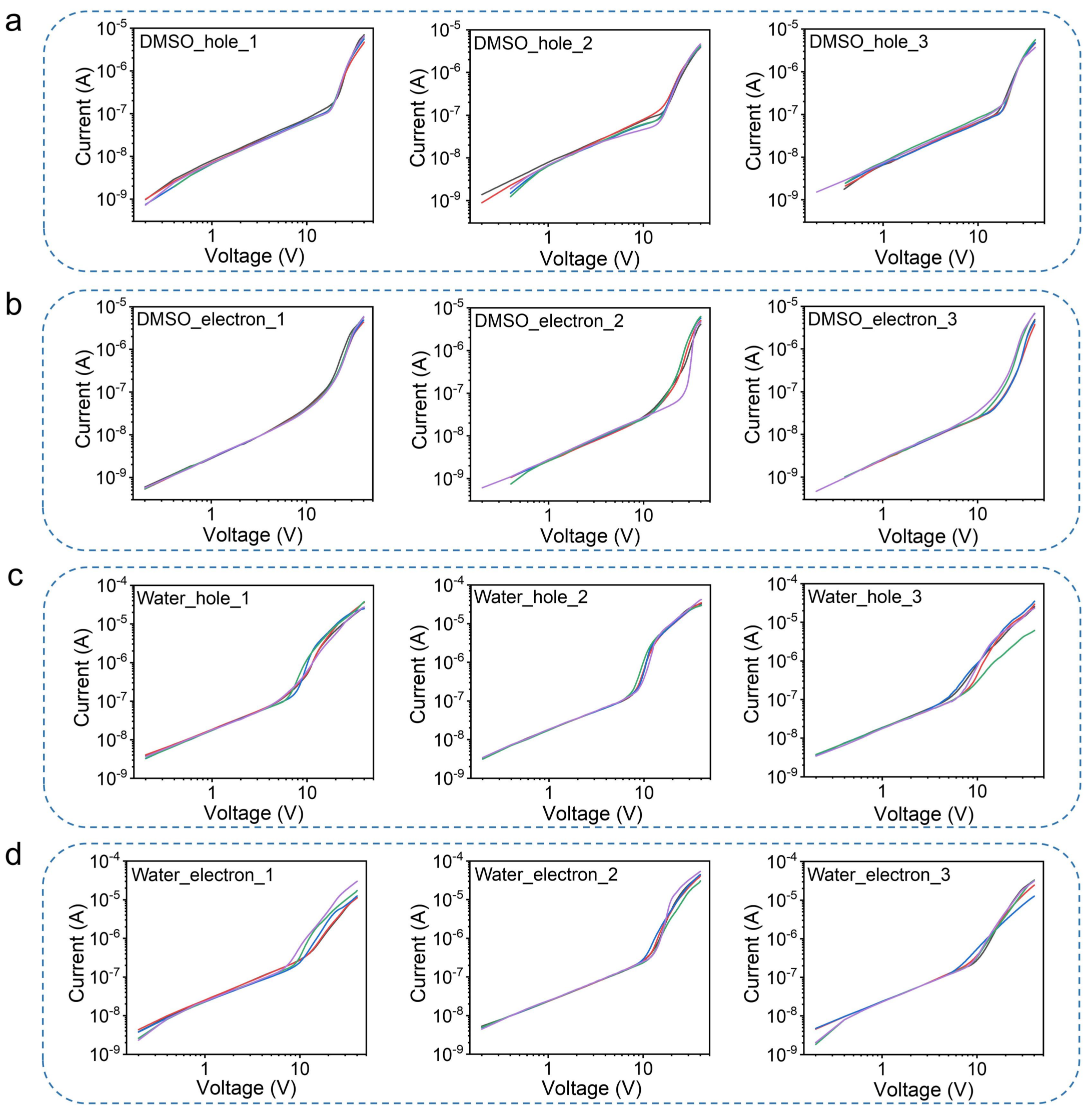

Supplementary Figure 13. Reproducibility of the *J-V* curves of (a) hole only devices based on DMSO-grown crystals, (b) electron only devices based on DMSO-grown crystals, (c) hole only devices based on water-grown crystals and (d) electron only devices based on water-grown crystals, obtained from three batches of devices with five continuous scans at room temperature.

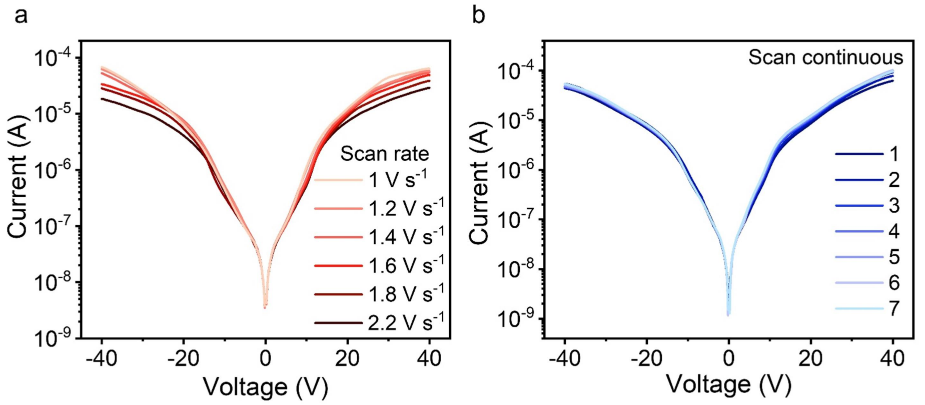

Supplementary Figure 14. (a) Typical scan rate and polarity dependent I-V curves of the $CsPbBr_3$ based devices and (b) I-V curves collected at the same scan rates for a number of continuous scans.

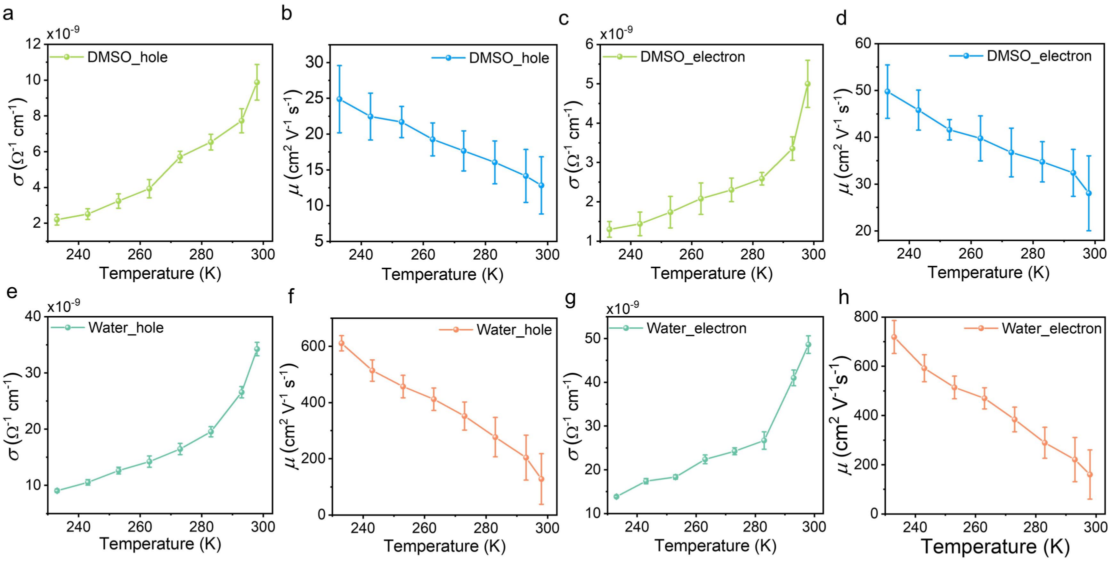

Supplementary Figure 15. Comparison of the calculated electrical properties based on the SCLC method. (a) Conductivity and (b) mobility of the hole-only device based on $CsPbBr_3$ SCs grown in DMSO, (c) conductivity and (d) mobility of the electron-only device based on $CsPbBr_3$ SCs grown in DMSO, (e) conductivity and (f) mobility of the hole-only device based on $CsPbBr_3$ SCs grown in water, (g) conductivity and (h) mobility of the electron-only device based on $CsPbBr_3$ SCs grown in water.

Supplementary Table 1. Comparison of the calculated electrical properties based on the SCLC measurements.

	DMSO_hole/electron			Water_hole/electron		
Temperature (K)	Conductivity ($\times 10^{-9}$ Ω^{-1} cm^{-1})	Trap density ($\times 10^{9}$ cm^{-3})	Mobility (cm^2 V^{-1} s^{-1})	Conductivity ($\times 10^{-9}$ Ω^{-1} cm^{-1})	Trap density ($\times 10^{9}$ cm^{-3})	Mobility (cm^2 V^{-1} s^{-1})
298	9.9/5.0	75.6/71.3	12.8/28.5	34.2/48.6	17.1/22.0	128/160
293	7.7/3.4	64.0/67.5	14.1/32.4	26.6/41.0	13.7/19.6	204/221
283	6.5/2.6	56.9/55.5	16.0/34.8	19.5/26.7	12.1/16.9	277/289
273	5.7/2.3	54.1/50.9	17.7/36.8	16.5/24.2	10.1/15.2	352/384
263	3.9/2.1	52.9/48.9	19.3/39.8	14.2/22.4	8.7/14.2	412/470
253	3.2/1.7	50.9/46.3	21.7/41.6	12.6/18.3	8.0/12.9	457/514
243	2.5/1.4	46.5/42.0	22.5/45.8	10.5/17.4	7.4/11.8	514/592
233	2.2/1.3	41.8/34.1	24.9/49.8	9.0/13.9	6.4/9.8	611/719

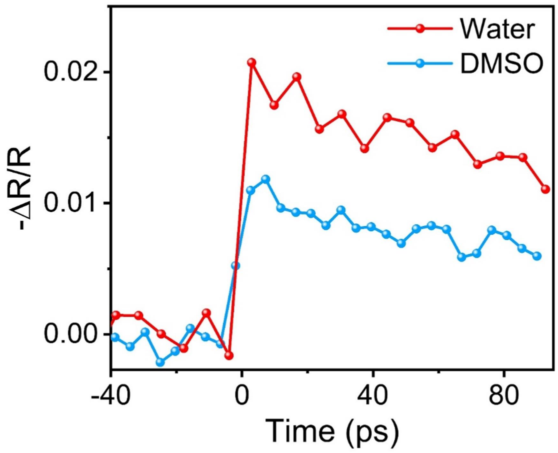

Supplementary Figure 16. Photoconductivity decay of the $CsPbBr_3$ SCs grown in DMSO and water, obtained via optical-pump-THz-probe spectroscopy (OPTPS) in a reflection geometry at room temperature. The SCs were photoexcited by ultrafast laser pulses at wavelength of 400 nm and fluence of 37 μJ cm^{-2}.

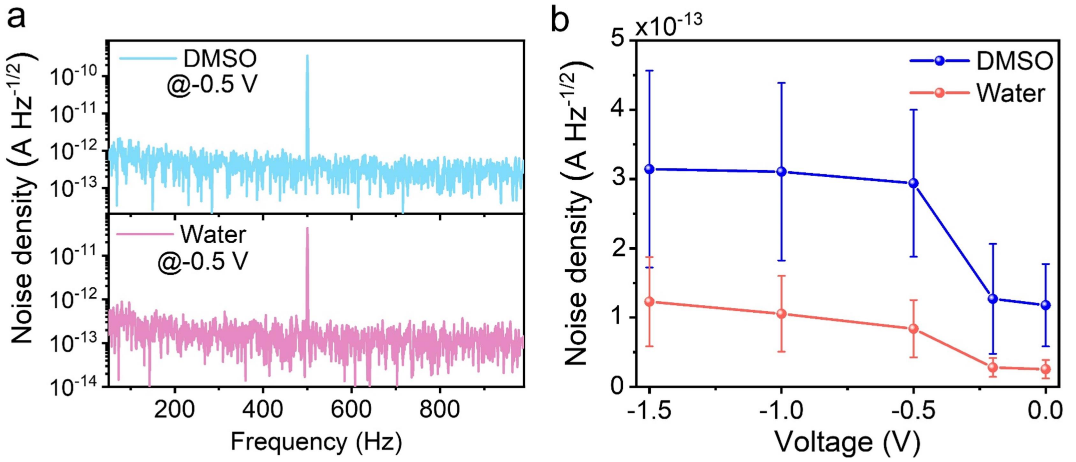

Supplementary Figure 17. (a) Noise spectra of the devices based on $CsPbBr_3$ SCs grown in DMSO and water under 500 Hz modulated weak light illumination (~3 nW cm^{-2}), (b) voltage dependent noise current measured at 1 kHz.

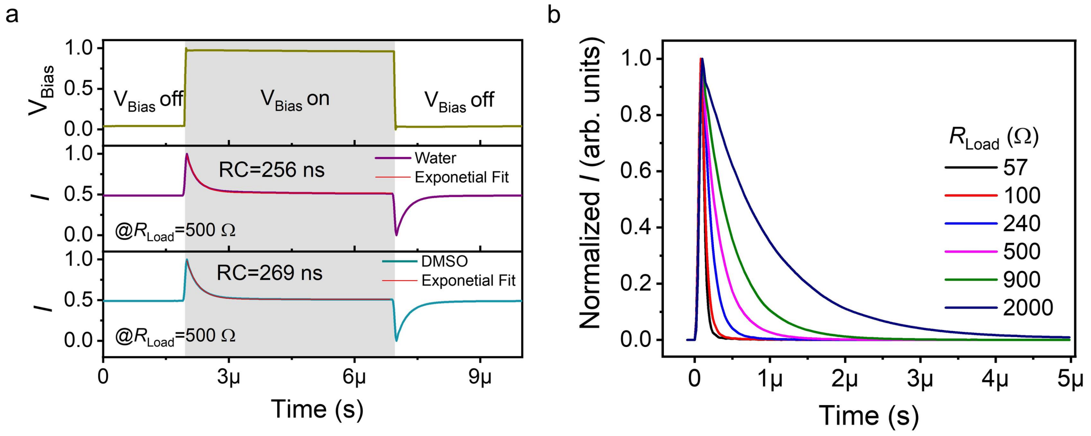

Supplementary Figure 18. (a) Recorded RC decay traces of the devices based on DMSO-grown and water-grown crystals, responding to the square-wave modulated bias voltage applied on these single crystal devices, and (b) load resistance dependent RC decay curves.

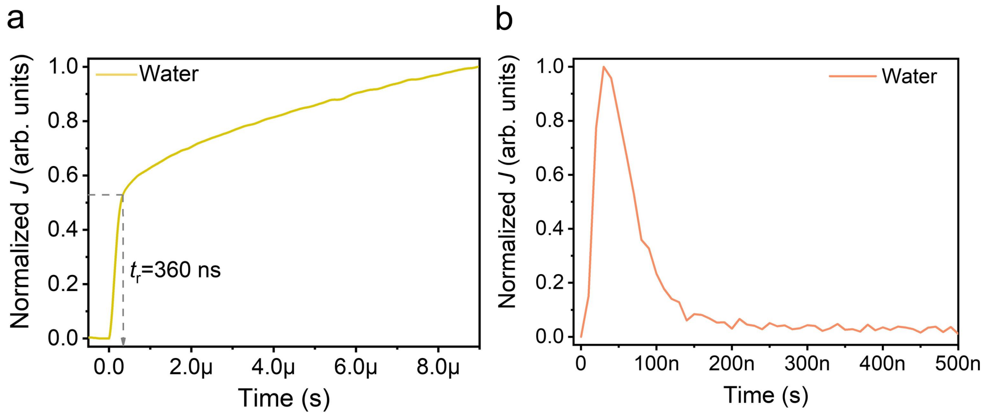

Supplementary Figure 19. The fast photocurrent transient of the detectors based on $CsPbBr_3$ SCs grown in water (a) under the illumination of a square-wave modulated 528 nm LED, and (b) a 10 ns laser shot.

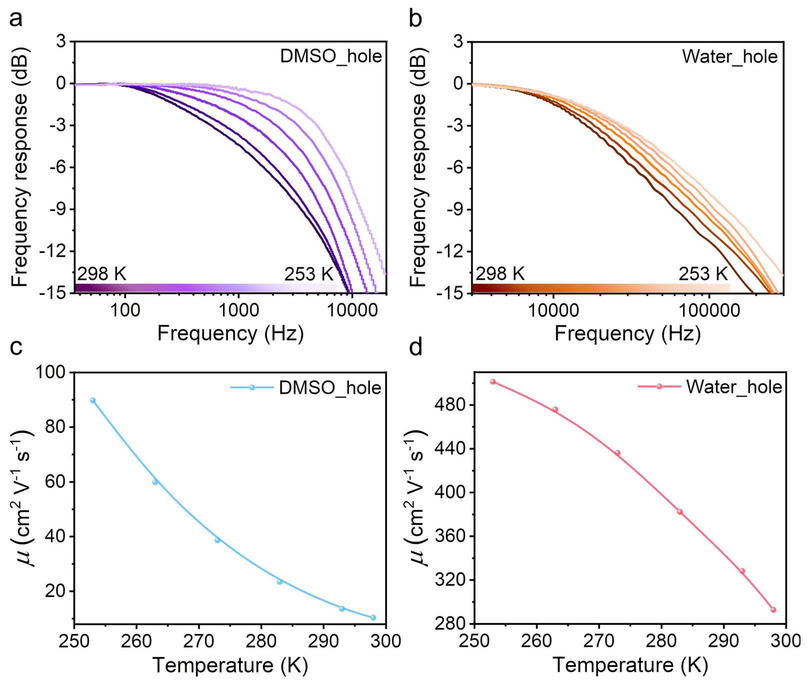

Supplementary Figure 20. Temperature-dependent frequency response of the devices based $CsPbBr_3$ SCs grown in (a) DMSO and (b) water, and the inferred carrier mobility of $CsPbBr_3$ SCs grown in (c) DMSO and (d) water.

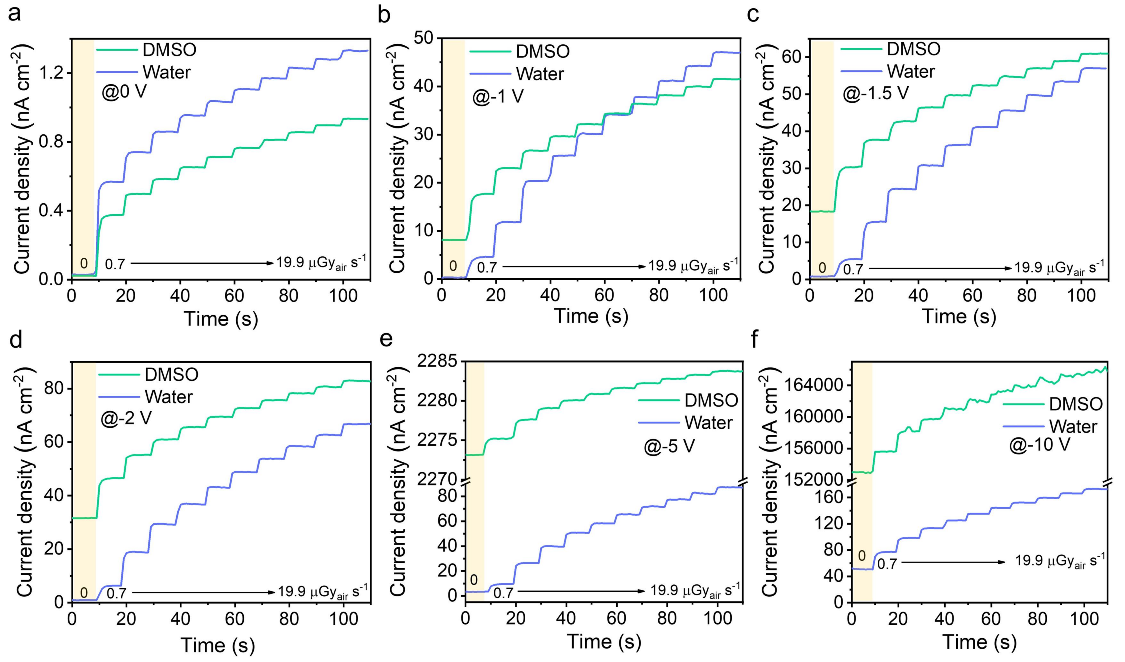

Supplementary Figure 21. Comparison of temporal response of the optimized X-ray detectors based on $CsPbBr_3$ SCs grown in DMSO and water at bias voltages of (a) 0 V, (b) -1 V, (c) -1.5 V, (d) -2 V, (e) -5 V and (f) -10 V, under various X-ray dose rates from 0 to 19.9 μGy_{air} s^{-1}.

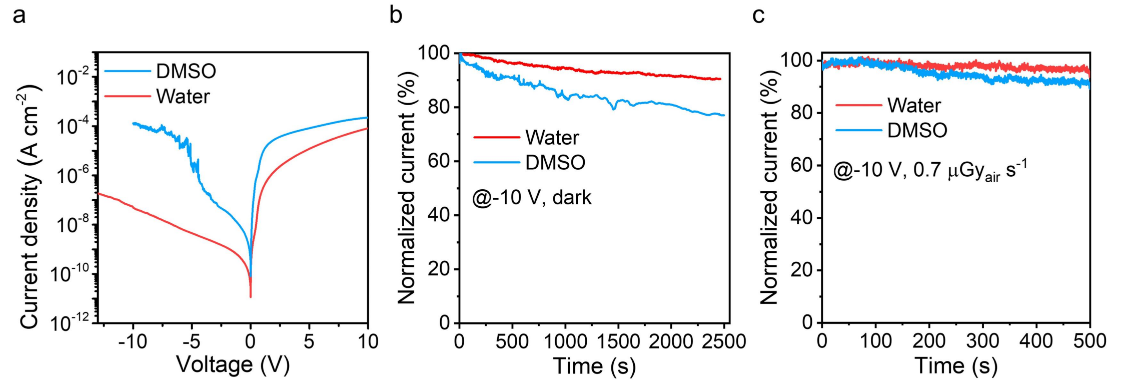

Supplementary Figure 22. Comparison of the (a) *J–V* curves measured in the dark with large bias voltage range, bias voltage stability at -10 V (b) in dark and (c) under X-ray illumination with a dose rate of 0.7 μGy_{air} s^{-1}.
